# The Plasma Glycome Differences Between Women with PCOS and Healthy Controls

**DOI:** 10.3390/ijms27052350

**Published:** 2026-03-03

**Authors:** Madison Holman, Sophie Jie Li, Mary M. Ahern, L. Renee Ruhaak, Siddika Karakas, Sridevi Krishnan

**Affiliations:** 1 Department of Ecology and Evolutionary Biology, School of Science, University of Arizona, Tucson, AZ 85721, USA; 2 Department of Physiology, College of Medicine, University of Arizona, Tucson, AZ 85721, USA; 3 School of Nutritional Sciences and Wellness, University of Arizona, Tucson, AZ 85721, USA; 4 Department of Clinical Chemistry and Laboratory Medicine, Leiden University Medical Center, 2333 Leiden, The Netherlands; 5 Department of Medicine, University of California Davis, Sacramento, CA 95616, USA

**Keywords:** PCOS, glycome, testosterone, tetraantennary glycans, hybrid-type glycans

## Abstract

While PCOS research has extensively explored genomic, transcriptomic, proteomic, and metabolomic milieus, our study examines the plasma glycome, comparing women with PCOS to age-matched healthy controls. In this observational study, n = 47 women with PCOS were screened and enrolled at the UC Davis Health campus; the comparator group constituted of n = 25 age-matched healthy women. During a study visit, body weight and body composition were measured, and fasted plasma samples were obtained to measure glucose, insulin, circulating lipids, and leptin, among other parameters, in both groups. In addition, in the PCOS group, circulating androgens and other endocrine hormones were measured. The plasma glycome was measured using a UHPLC-MS protocol. As expected, women with PCOS had higher body weight (*p* < 0.01), body fat (*p* = 0.004), fasting leptin (*p* = 0.01), insulin (*p* = 0.003), and glucose (*p* = 0.004). Hybrid-type glycans were reduced (*p* = 0.019), while tetraantennary (glycans with four branches) were modestly increased (*p* = 0.05) in women with PCOS compared to healthy controls. SVEM–LASSO (bootstrapped) regression models further supported a higher tetraantennary and lower hybrid glycan profile as representative of women with PCOS (AUROC: 0.81, accuracy: 82), even when adjusted for body weight (AUROC: 0.89, accuracy: 80) and body fat mass (AUROC: 0.89, accuracy: 86). Furthermore, in women with PCOS, total testosterone was positively correlated with tetraantennary glycans (r = 0.322, *p* = 0.029). We report novel findings of elevated tetraantennary and reduced hybrid-type N-glycans in PCOS, and a potential association between circulating androgens and protein glycosylation. Given the pilot nature of this study, larger cohort investigations are required to validate these observations.

## 1. Introduction

Polycystic ovary syndrome (PCOS) is a complex endocrine disorder affecting one in 10 women of reproductive age, characterized by reproductive manifestations such as oligomenorrhea/oligo-ovulation or amenorrhea and androgen excess, as well as metabolic manifestations such as insulin resistance, with symptoms varying across a spectrum [1,2]. There is currently no established root cause for the imbalance of hormones, and, subsequently, no cure; treatment plans primarily address symptoms [2]. Since relatively small amounts of weight loss (5–7%) and /or treatment of insulin resistance improve reproductive function in PCOS, there is likely a functional link between the two facets of this disorder. The ability of modest weight loss (5–7%) and insulin-sensitizing treatments to improve reproductive function in PCOS emphasizes the central role of metabolic dysregulation in driving reproductive abnormalities, a relationship further supported by emerging genetic, metabolomic, and lipidomic evidence.

Research employing “omic” technologies has contributed to the understanding of PCOS, providing valuable insights into the molecular pathways involved [3]. Genomic investigations have demonstrated that PCOS is strongly heritable [4] and polygenic [5], and genome-wide association studies have identified susceptibility loci and candidate genes for PCOS that, unsurprisingly, are associated with insulin signaling, glucose homeostasis, and skeletal muscle homeostasis [6,7,8,9,10,11]. The involvement of gene-mediated extraovarian androgen actions in PCOS further suggests that it is not solely an ovarian disorder [12,13]. Follicular fluid lipidomics has identified several ceramides, free fatty acids, triglycerides, phosphatidylethanolamines, and phosphatidylinositols as increased in women with PCOS compared to controls [14,15], with some overlap in ceramide profiles between insulin resistance and PCOS [14]. Gut microbiota and metabolomic studies have also identified that Clostridiales, Bacteroidetes, and Porphyromonadaceae differ in people with PCOS compared to healthy controls [16,17,18,19,20].

The N-glycome has, in recent years, emerged as a critical component of metabolic health, yet its role in many complex disorders remains insufficiently explored [21]. The N-glycome comprises all sugars, glycans, or oligosaccharides added to proteins in post-translational or co-translational processes [22]. N-glycans have both structural and functional roles, the disruption of which can adversely affect homeostasis. In particular, sialylation (the addition of the nine-carbon sialic acid sugars to proteins), fucosylation (the addition of five-carbon fucose), and branching (increased bisecting N-acetylglucosamines -GlcNAcs and/or antennae) have been found to profoundly influence immune and inflammatory diseases, cancer progression, and metabolic disease onset [23,24,25,26]. Despite its established relevance to metabolic and inflammatory pathways, the N-glycome has not been investigated in PCOS. Because N-glycan alterations can both drive and reflect disease processes [21], this absence of glycomic profiling represents a critical gap in understanding PCOS. Addressing this gap may yield novel insights into disease mechanisms and potential biomarkers.

Here we present results from our pilot investigation, a first of its kind, into differences in the plasma N-glycome of women with PCOS compared to age-matched healthy controls. Since the influence of sex-steroid hormones (estrogens and androgens) on the glycosylation process is poorly understood [27], it is difficult to ascertain whether observed changes are causal or reactive. However, this study represents the first step in furthering our understanding of any differences that do exist.

## 2. Results

### 2.1. Clinical Characteristics of Study Participants

A total of 72 women were included in this study: 47 with PCOS and 25 healthy controls. Differences in demographic and clinical outcomes are presented in Table 1. Women with PCOS had higher BMI but similar age compared with controls. Not surprisingly, they exhibited greater fat mass, lean mass, higher CRP, fasting insulin, glucose, leptin, and triglycerides. By contrast, adiponectin and HDL-c were lower in PCOS. No significant difference was observed in total cholesterol between groups.

### 2.2. Dimension Reduction Was Used to Identify Glycomic Variables for Non-Parametric Testing

**Figure 1:**
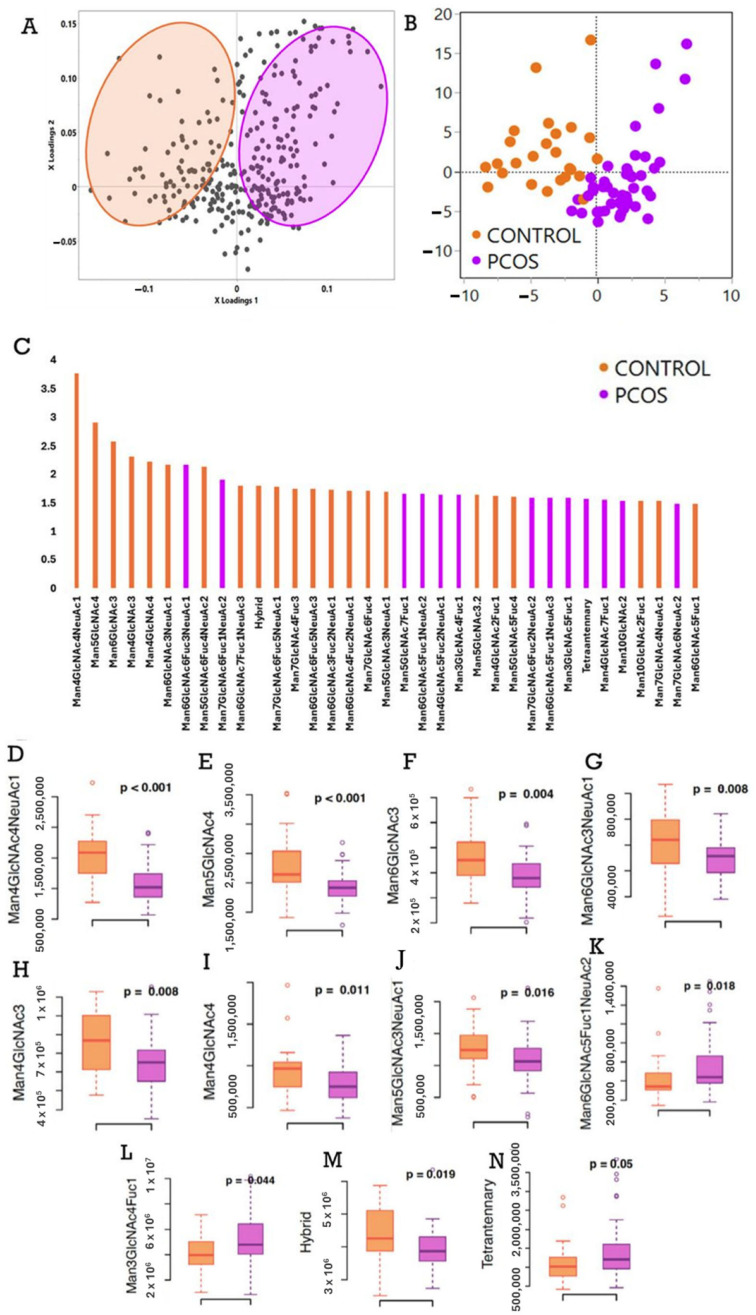
The plasma glycome differences between PCOS and healthy controls. (**A**) The loadings plot of the partial least squares discriminant analysis comparing the plasma glycome between women with PCOS (purple) and healthy controls (orange), each gray circle represents one glycan species. (**B**) The scores plot—women with PCOS are purple and healthy controls are orange. (**C**) The VIP (variable importance in projection) plot, where the Y-axis shows the VIP score and the X-axis shows the individual or summed glycans that are the strongest discriminators between women with PCOS and healthy controls in this identified model. (**D**–**N**) Significantly different individual and summed glycans between women with PCOS (depicted in purple) and healthy controls (orange). Of note, women with PCOS had lower plasma hybrid glycans and higher tetraantennary glycans compared to healthy controls.

The PLS-DA model that provided the best discrimination between PCOS and control group showed modest separation, with a Q^2^ value of 0.37, two factors with a root mean PRESS value of 0.791, R^2^X of 0.2 (20%), and R^2^Y of 0.856 (85.6%). VIP variables from this model with scores > 1.5 were used in subsequent non-parametric tests (see Figure 1A–C).

### 2.3. Glycan Trait Differences Between Healthy Controls and Patients with PCOS

Women with PCOS had a significantly lower concentration of hybrid glycans (*p* = 0.019) than healthy controls. There was also a moderately significant difference (*p* = 0.05) in tetraantennary glycans—higher in women with PCOS compared to healthy controls (Figure 1M,N).

### 2.4. Relationships Between Plasma Glycome and Anthropometric, Endocrine, and Metabolic Parameters

**Figure 2:**
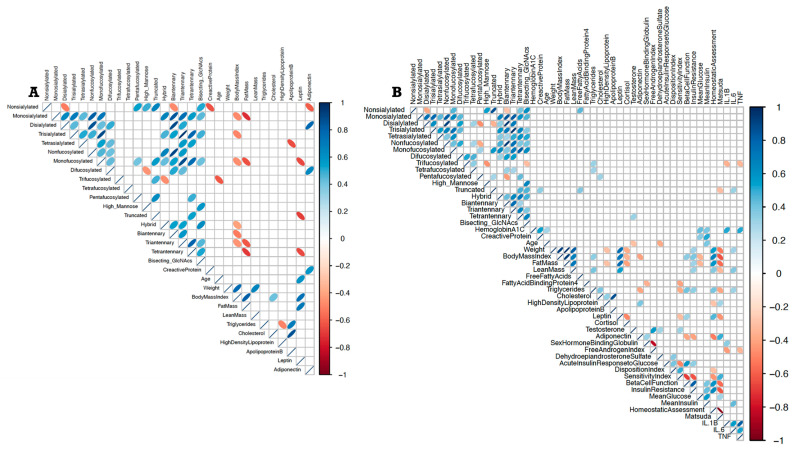
Correlogram of glycomic, anthropometric, clinical, and metabolic features of healthy controls (n = 25) (**A**) and women with PCOS (n = 47) (**B**). Significant positive associations (based on Spearman’s rho) are depicted by blue ellipses and inverse associations by orange/red ellipses. Light vs. dark shades of same color indicate weak vs. strong associations. Thick vs. thin ellipses (if it almost resembles a line) represent wider vs. tighter 95% confidence intervals around the scatter area. Women in the healthy control group display positive relationships between leptin and oligomannose-rich glycans, as well as between sialylated and fucosylated glycans with adiponectin, while these relationships are either weaker in women with PCOS or non-existent.

The PCOS group had more endocrine and clinical information than the control group, resulting in a larger correlogram (Figure 2). In PCOS, testosterone was positively associated with tetraantennary glycans (r = 0.322, *p* = 0.029). Fasting triglycerides correlated positively with higher-order fucosylated glycans (trifucosylated: r = 0.321, *p* = 0.027; tetrafucosylated: r = 0.303, *p* = 0.038). CRP and IL-6 were positively associated with truncated glycans (r = 0.337, *p* = 0.026; r = 0.329, *p* = 0.032), whereas the Matsuda index (OGTT-derived) was inversely associated with truncated glycans (r = −0.307, *p* = 0.036).

In the control group, leptin (and fat mass) was inversely associated with tetraantennary (r = −0.650, *p* = 0.026), truncated (r = −0.685, *p* = 0.017), and mono-fucosylated glycans (r = −0.657, *p* = 0.024). Adiponectin was inversely associated with non-sialylated glycans (r = −0.608, *p* = 0.040) and positively associated with di-sialylated (r = 0.783, *p* = 0.004) and di-fucosylated glycans (r = 0.643, *p* = 0.028). No significant associations between leptin or adiponectin and the plasma glycome were observed in PCOS.

### 2.5. Glycomic Predictors of PCOS vs. Controls, Adjusted for Body and Body Fat Mass

**Figure 3:**
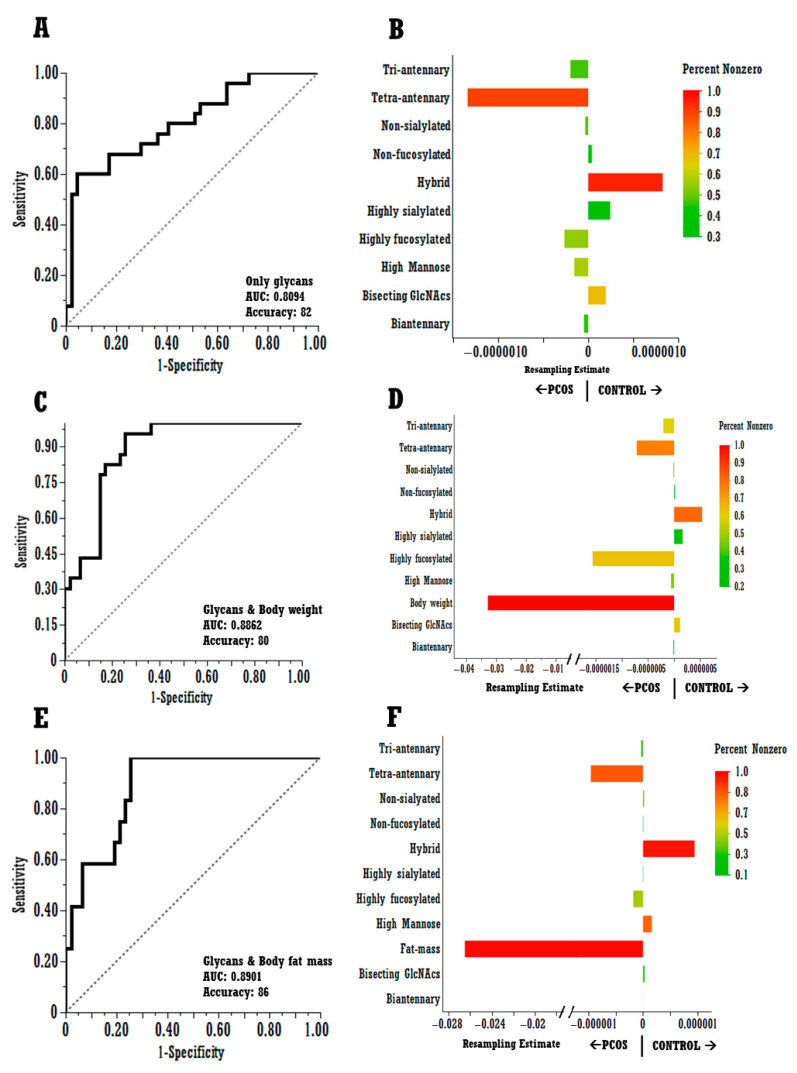
Self-validated ensemble model–LASSO analysis to evaluate the role of summed glycan traits in predicting PCOS vs. control groups, and the addition of body weight and adiposity to these models to assess how that changes which glycomic predictors remain. Panels (**A**,**C**,**E**) show AUC ROC with accuracy and indicate which predictor variables and confounders were used. Panels (**B**,**D**,**F**) show resampling estimates (i.e., coefficients) on the X-axis and the features (i.e., glycome or body weight/fat) on the Y-axis. Bars show the average SVEM–LASSO coefficient for each feature (X-axis, roughly centered at 0), with positive values indicating higher levels in controls and negative values indicating higher levels in PCOS. The color scale (red–yellow–green) indicates the percentage of bootstrap fits in which the feature’s coefficient was non-zero (selection probability).

We used SVEM (bootstrapped)–LASSO-penalized regression models to evaluate the predictive capability of glycan traits to classify PCOS and healthy controls, and to identify whether the glycome differences we observed between PCOS and control groups of women are primarily driven by body weight/fat mass. Figure 3 represents the outcomes of these models. When glycan traits were used to predict PCOS and control groups, hybrid and tetraantennary glycans were identified as significant predictors of control and PCOS groups, respectively, with body weight (AUROC: 0.87, accuracy: 80), body fat mass (AUROC: 0.89, accuracy: 86) and without any confounders (AUROC: 0.81, accuracy: 82). The percent non-zero for hybrid glycans was 95, 87, and 95 in glycans-only, glycans + body weight and glycans + body fat mass adjusted models, respectively, and the percent non-zero for tetraantennary glycans were 91, 70, and 74.5, respectively (See Supplemental Table S2). The consistently >70 percent non-zero estimate for both hybrid and tetraantennary glycans indicates they are selected in a large majority of resamples and thus behave as stable predictors rather than artifacts of one particular split. Thresholds in the 60–90 range are commonly used to define “stably selected” features [28,29]. Furthermore, the fact that hybrid glycans’ percent non-zero estimates barely drop and remain in the 80s and 90s, even when adjusting for body weight and body fat mass, suggests a strong independent predictive ability not driven by these confounders. Tetraantennary glycans, on the other hand, drop from 91 to 70 when adjusted for body weight and fat mass, suggesting that their association with PCOS–control status is shared with confounders, while still retaining an independent contribution across resamples in this dataset. As evident in these models, body weight and fat mass are strong predictors of the PCOS group as well.

## 3. Discussion

This pilot cross-sectional comparative study of the plasma glycome identified tetraantennary glycans to be elevated and hybrid glycans to be lower in the PCOS group compared to the healthy controls. This glycomic profile appears independent of body weight or fat mass, although some of the relationship between tetraantennary glycans and PCOS could be mediated by body adiposity, unlike hybrid-type glycans. Furthermore, the positive association between highly branched glycans and testosterone, one of the primary androgens involved in precipitating the ovarian disruptions observed in PCOS, suggests that further research should explore the possibility of the ratio of branched to hybrid glycans as detection biomarkers, at least for certain phenotypes of PCOS.

Tetraantennary glycans, characterized by their branched structure with four antennae, play a significant role in various biological processes, including metabolic diseases and inflammation [30]. Mechanistically, tetraantennary N-glycans reflect high MGAT5 (N-acetylglucosaminyltransferase V) activity and hexosamine flux, which in vivo tend to favor fat accumulation and weight gain under calorie-rich conditions [31]. MGAT5-driven β1,6-GlcNAc branching supports tri-/tetraantennary N-glycan biosynthesis and promotes fat accumulation in mouse and cell models [32]. Perhaps due to this, highly branched N-glycans (MGAT5 products) are a recognized feature of chronic inflammatory states and are upregulated in several gynecologic and systemic diseases [33]. Because MGAT5-dependent N-glycan branching promotes nutrient uptake, lipid storage, and hormone receptor signaling in other metabolic and inflammatory contexts [34], MGAT5 and its tetraantennary glycan products are plausible contributors to PCOS pathophysiology; however, this has not yet been directly tested in PCOS models or patient cohorts. In the data presented here, tetraantennary glycans show robust predictive ability for classifying PCOS and healthy controls. However, the attenuation but persistence of this association after adjustment for body weight/body fat mass suggests that tetraantennary glycans may partially mediate obesity-related risk in PCOS.

The synthesis of glycans is mediated by a series of glycosyltransferases that add specific sugar residues to growing glycan chains. When more branched glycans, such as tetraantennary or highly branched complex glycans, are synthesized, they may compete for the same substrates and enzymes that are responsible for producing hybrid glycans [35]. The presence of additional sialic acid residues and branching can enhance the retention of certain glycans, potentially leading to the exclusion of hybrid glycans from the glycan pool [36]. Furthermore, the presence of branched structures can enhance the binding affinity of certain glycan-binding proteins, indicating that the glycan structure can influence enzymatic pathways and potentially reduce the synthesis of hybrid-type glycans [37]. Via such mechanisms, a milieu that promotes more tetraantennary glycans could influence the glycome to container fewer hybrid-type glycans. Hybrid-type glycans play critical roles in fertility. Reductions in hybrid-type glycans in oocytes have been shown to reduce their ability to be fertilized [38]. Furthermore, oocytes lacking hybrid-type glycans do not mature past embryonic day 9.5 during fertilization [39,40]. Thus, a systemic circulatory milieu that has more tetraantennary and lower hybrid glycans could be involved in and reflect the metabolic and fertility disruptions seen in women with PCOS.

In the current study, we observed a positive association between tetraantennary glycans and testosterone in the PCOS group. Elevated testosterone, a hallmark of PCOS, is associated with various metabolic disturbances, including insulin resistance, dyslipidemia, and increased risks for type 2 diabetes and cardiovascular disease [41,42]. Insulin resistance has been linked to changes in glycosylation patterns of plasma proteins in women with gestational diabetes mellitus [43]. Specifically, insulin resistance is associated with a decrease in low-branched glycans and an increase in high-branched glycans, such as tetraantennary glycans, on plasma proteins [43]. This shift in glycosylation patterns is also typically observed in type 2 diabetes, which can be a consequence of insulin resistance. In men, low testosterone levels are associated with metabolic syndrome, type 2 diabetes, and obesity, and testosterone replacement therapy has been shown to improve glycemic control and insulin resistance [44,45,46]; however, its effects on protein glycosylation have not been investigated. Moreover, the impact of testosterone on glycosylation in women remains unknown. Future studies should examine the relationships between circulating estrogens, androgens, and protein glycosylation to establish a foundational understanding of the “endocrine–glycome” axis.

### Limitations and Future Directions

The current study is a cross-sectional investigation and is only able to report observational differences in a small sample size between healthy and women with PCOS, and associated metabolite relationships. Furthermore, the lack of testosterone and a few other clinically critical measures in the control group made a complete comparison not possible. The choice to not include multiple comparison corrections and the modest discriminatory ability of the presented PLS-DA model also warrant cautious interpretation of the results.

However, no study to date has evaluated the plasma glycome in women with PCOS, thus contributing to the novelty of this manuscript. A higher tetraantennary and lower hybrid-type glycan plasma profile in women with PCOS suggests the presence of a pro-inflammatory metabolic disease-like milieu. While this is not surprising, it lends itself to future investigations that can identify whether these glycans can be used to better detect PCOS phenotypes, which can then inform appropriate therapeutic approaches.

## 4. Materials and Methods

This report includes a cross-sectional comparison of plasma glycome in women with PCOS and a control group of healthy women.

### 4.1. Participants

This study utilized previously obtained anthropometric, biochemical, and clinical data, along with stored plasma samples from investigations [47,48,49] that were approved by the Institutional Review Board of the University of California, Davis. All participants signed written informed consent, and all research was carried out in accordance with the World Medical Association Declaration of Helsinki.

Both the control and PCOS groups consisted of women aged 20–45 years with a BMI of 25–45 kg/m^2^. We age-matched women with and without PCOS to ensure they were at similar life stages. We did not BMI-match because this would not reflect the real-world distribution of BMI among women with PCOS. Additionally, adiposity might amplify and mediate the metabolic risk associated with PCOS [50], and matching on BMI could remove clinically relevant differences.

The PCOS group fulfilled the NIH diagnostic criteria for PCOS, requiring ovarian dysfunction (amenorrhea: >6 months without menstruation, or oligomenorrhea: <6 periods per year), clinical or biochemical evidence of hyperandrogenemia (total testosterone >54 ng/dL or free testosterone >9.2 pg/mL), and absence of confounding conditions (e.g., Cushing’s disease, 21-hydroxylase deficiency, or prolactinoma). Exclusion criteria included recent use (within two months) of drugs including oral contraceptives, anti-androgenic medications, insulin sensitizers, or lipid-lowering drugs, as well as diabetes, untreated thyroid disorders, systemic illnesses (renal, hepatic, or gastrointestinal), smoking, or consumption of more than two alcoholic drinks per week.

Data collection occurred at the Clinical and Translational Science Center Clinical Research Center (CCRC) at the University of California, Davis. Women with regular menstrual cycles were tested during the early follicular phase of the cycle. Patients who were not cycling were tested randomly, as they were anovulatory (in women with chronic anovulation, a reliable follicular-phase window cannot be identified, and the typical mid-cycle surge and luteal progesterone rise are absent, resulting in less predictable, phase-dependent hormonal fluctuations; thus, day-specific timing is less critical) [51].

All participants provided an overnight fasted blood sample via venipuncture, which was processed, aliquoted, and stored at −80 °C until analysis.

### 4.2. Anthropometric Data

The Tanita BWB800-P Digital Medical Scale was used to measure body weight in light clothing when patient came in for a clinic visit (once, in the early follicular phase for regularly menstruating women, and any time for anovulatory women). During the same visit, body composition was measured using bioelectrical impedance 13 (Biostat Co., Isle of Man, UK) [52].

### 4.3. OGTT—Only in Women with PCOS

After an overnight fast, baseline samples were obtained; participants drank 75 g of glucose (Glucola™), and additional blood samples were obtained every 30 min for 2 h.

### 4.4. Biochemical Measurements

Fasting glucose was measured using YSI 2300 STAT Plus Glucose & Lactate Analyzer (YSI Life Sciences, Yellow Springs, OH, USA). Fasting insulin was measured by RIA (Milipore, St. Charles, MO, USA). To evaluate insulin function, various insulin resistance and sensitivity indices were calculated (HOMA-IR, HOMA-B, Matsuda index, disposition index, sensitivity index, mean glucose and insulin measures [53]). Triglycerides, cholesterol, and HDL-cholesterol were measured using the Poly-Chem System Analyzer (Cortlandt Manor, NY, USA). Leptin and adiponectin were measured using RIA (Millipore, St. Charles, MO, USA). hs-CRP was measured using a highly sensitive (hs) latex-enhanced immunonephelometric assay. Total testosterone, sex hormone-binding globulin (SHBG), and DHEAS were measured by RIA (Diagnostic Systems Laboratories, Webster, TX, USA).

### 4.5. N-Glycan Preparation

All glycome analyses were completed between 2012 and 2013 on samples stored at −80 °C. N-glycans were released from plasma as previously described [54,55]. In brief, N-glycans were prepared from 50 µL of human plasma after protein denaturation with 50 μL of 200 mmol/L ammonium bicarbonate (Sigma-Aldrich, St. Louis, MO, USA) solution with 10 mmol/L dithiothreitol (Promega, Madison, WI, USA). A total of 2 µL of PNGase F (New England Biolabs, Ipswich, MA, USA) was added to the samples, and glycans were enzymatically released in a CEM microwave, followed by purification and enrichment by solid-phase extraction using PGC on a 96-well plate. Released N-glycans were dried in vacuo prior to analysis.

### 4.6. nHPLC-chipTOF-MS Analysis

The dry N-glycan sample was reconstituted in 225 μL of water and transferred to an injection vial for analysis; 1 μL of the sample was used for injection. N-glycans were analyzed using an Agilent nanoHPLC-chip-TOF-MS, in which a PGC chip II (Agilent Technologies, Santa Clara, CA, USA) packed with porous graphitized carbon was used. Upon injection, the sample was loaded onto the enrichment column and subsequently separated on the analytical column using a gradient from 3% acetonitrile (ACN) with 0.5% formic acid (FA) (solvent A) to 90% ACN with 0.5% FA (solvent B). The mass spectrometer was operated in positive mode.

### 4.7. Data Analysis

Data analysis was performed using Masshunter^®^ (Version B.05.12) qualitative analysis according to Hua et al. [56], with modifications. Data were loaded into Masshunter^®^ qualitative analysis, and glycan features were identified and integrated using the Molecular Feature Extractor algorithm (Version 10.2). A retrosynthetic theoretical glycan library containing 331 possible N-glycan compositions was used for glycan identification [57]. To increase biological relevance, similar types of glycans were grouped, and summations were calculated. For instance, all glycans with a single sialic acid were summed into a category labeled monosialylated glycans. This procedure was repeated for sialylations and fucosylations. In addition, glycans were classified and summed based on their structure (see Supplemental Table S1).

### 4.8. Statistical Analysis

Data analyses were performed using R Version 2023.06.1 [58] and JMP Pro 18.1 (SAS Institute, Cary, NC, USA). Data missingness was evaluated using the Amelia package in R [59]. The missingness map (Supplemental Figure S1) indicated that 12% of all data (and 28% of clinical data) were missing. A significant portion of the clinical data were missing only for controls, as these measurements were not obtained. Because missing data could not be considered missing at random, data imputation was not conducted. Final analyses were conducted using complete-case data [60]. Data normality was evaluated using Q-Q plots, Shapiro–Wilk tests and Kolmogorov–Smirnov tests. All data were normalized for further analyses (log transformation for some clinical variables and Johnson transformation for the remaining clinical and all glycome variables), and non-parametric tests were used where appropriate.

Given the wide and short dataset (n << parameters), a partial least squares discriminant analysis (PLS-DA) was used for dimension reduction with all individual glycans and summated glycan subtypes to identify their ability to discriminate between the PCOS and control groups. For the PLS-DA, normalized data were used in a NIPALS algorithm, employing leave-one-out cross validation (due to the small dataset and computational efficiency), with a VIP (variable importance in projection) score of >1.5 used as a cut-off to deem a parameter strong enough to discriminate between PCOS and controls. Wilcoxon tests were used to determine whether there were significant differences in selected glycomic variables and clinical parameters between women with PCOS and healthy controls; *p*-values < 0.05 were considered significant. To preserve power in this pilot study, given the modest number of comparisons (and the use of PLSDA as a dimension reduction tool), we did not formally adjust for multiple testing. Instead, exact *p*-values are reported, and these findings require independent replication in larger cohorts [61]. Spearman’s rho tests were used to evaluate correlations between selected glycomic and clinical parameters for the PCOS and control groups.

Self-validated ensemble model (SVEM)–LASSO with leave-one-out cross-validation was used to further investigate the predictive ability of summed glycans in classifying PCOS and healthy controls. These models were also used to evaluate the effect of adding or removing body weight and body composition parameters on the predictive ability of glycans in classifying PCOS and healthy controls. SVEM–LASSO models, in addition to being penalized regressions, are better suited for small, noisy omics datasets than a single ordinary LASSO fit [62] due to the use of stability criteria [63]. Briefly, SVEM–LASSO repeatedly fits LASSO models (here, 200 fractional-weight bootstraps) to slightly reweighted, anti-correlated versions of the same dataset, each time using one weight column as “training” and the paired anti-correlated weight column as “validation”, so that every observation contributes to both fitting and testing the model. The resulting parameter estimates from these fits are averaged into a single ensemble, yielding a penalized model that has been internally validated [62]. Results from the SVEM–LASSO model are presented as an ensemble of selected predictors with their average coefficients (and directions of association), along with overall performance metrics such as AUROC and accuracy estimated from the internal self-validation procedure. The stability of variable selection (i.e., the percentage of times each variable has a non-zero coefficient across ensemble fits) was also used to identify which predictors are robust candidates in this dataset.

## Figures and Tables

**Table 1:** Clinical data of the control and PCOS groups.

Parameter	Control (n = 25)	PCOS (n = 47)	*p*-Value	Reference Ranges
Age (y)	30.1 ± 8.1	32.0 ± 6.3	0.610	
CRP (mg/L)	1.8 ± 1.3	6.0 ± 7.1	**0.020**	<3 mg/L
Weight (kg)	69.8 ± 7.7	97.1 ± 20.9	**<0.01**	
BMI (kg/m^2^)	25.7 ± 3.5	35.5 ± 7.9	**<0.01**	<24.9 kg/m^2^ *
Fat-mass (kg)	27.5 ± 4.8	44.0 ± 16.3	**0.004**	
Lean mass (kg)	46.0 ± 4.5	53.2 ± 5.5	**0.003**	
Triglycerides (mg/dL)	83.5 ± 32.3	107.5 ± 51.0	0.072	<150 mg/dL
Total Cholesterol (mg/dL)	177.6 ± 32.6	186.6 ± 35.5	0.098	<200 mg/dL
HDL-c (mg/dL)	50.3 ± 11.6	40.2 ± 9.3	**<0.01**	>50 mg/dL
Apolipoprotein-B (mg/dL)	62.5 ± 22.5	76.7 ± 19.8	**0.039**	60–117 mg/dL
Leptin (ng/mL)	15.2 ± 6.1	24.9 ± 12.9	**0.010**	0.5–15.2 ng/mL
Adiponectin (μg/mL)	12.9 ± 5.4	9.6 ± 5.6	**0.033**	4–37 μg/mL
Fasting insulin (mIU/mL)	11.9 ± 3.5	20.4 ± 9.8	**0.003**	<20 mIU/mL
Fasting glucose (mg/dL)	90.1 ± 7.7	98.1 ± 12.3	**0.004**	<100 mg/dL
Total Testosterone (ng/mL)		0.86 ± 0.28	-	0.15–0.7 ng/mL

Data are presented as mean ± standard deviation. BMI, body mass index; CRP, C-reactive protein; HDL-c, high-density lipoprotein cholesterol. *p*-values indicate comparisons between PCOS and healthy controls. * BMI of <25 is considered normal weight. Bolded *p*-values indicate significant differences between Controls and PCOS.

## Data Availability

All glycome data will be made available upon reasonable request. We will share data on our GitHub page: https://github.com/Glyconutlab.

## Supplementary Materials

The following supporting information can be downloaded at: https://www.mdpi.com/article/10.3390/ijms27052350/s1.
