# Supplementary material for: The Plasma Glycome Differences Between Women with PCOS and Healthy Controls
Source: Int J Mol Sci. 2026 Mar 3;27(5):2350. doi: 10.3390/ijms27052350 (PMC12985897; doi:10.3390/ijms27052350)
Supplement: Supplementary file 1 [file ijms-27-02350-s001.zip › Supplemental Figure S1.pdf]

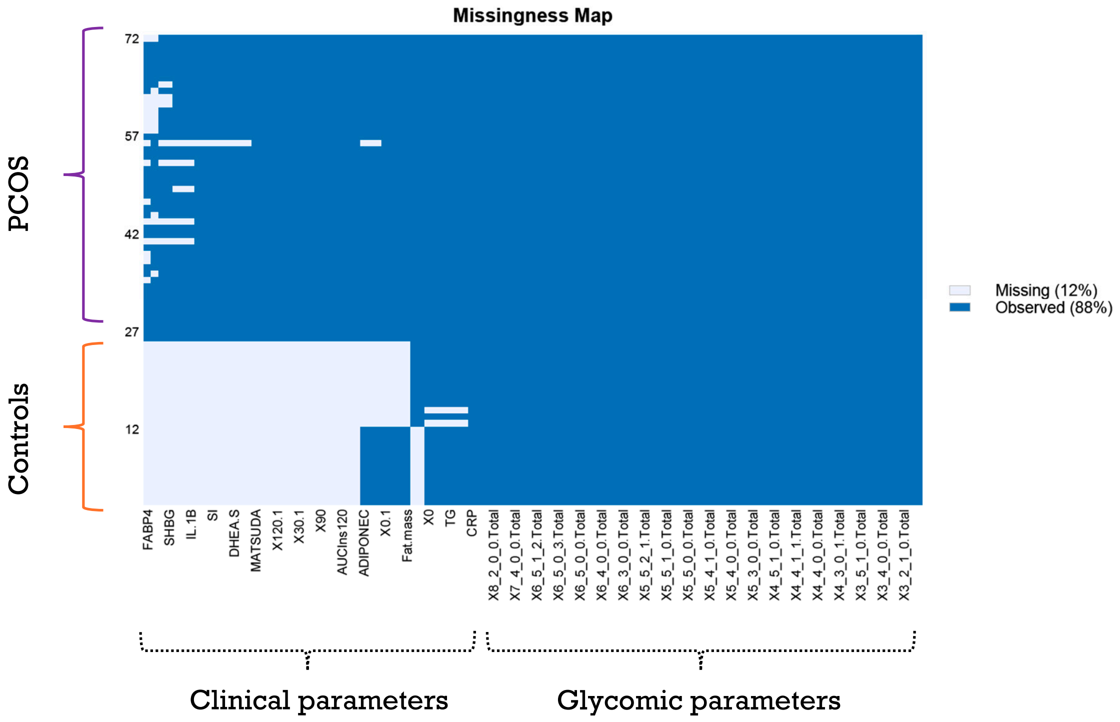

Supplemental Figure 1: Missingness map from Amelia package in R showing 12% of data missing, and most of it in clinical parameters and in the control group.
