# Supplementary material for: The Plasma Glycome Differences Between Women with PCOS and Healthy Controls
Source: Int J Mol Sci. 2026 Mar 3;27(5):2350. doi: 10.3390/ijms27052350 (PMC12985897; doi:10.3390/ijms27052350)
Supplement: Supplementary file 1 [file ijms-27-02350-s001.zip › supplementary Table S2.pdf]

Supplemental Table S2: Composite tables of SVEM- Lasso regression models (LOO cross validation) showing resampling estimates (average across 200 bootstraps), standard deviations and % of times that variable was a non-zero estimate (stability).

Only Glycomic features

Term	Resampling Estimate	Resampling Std Dev	Percent Nonzero
Intercept	-3.810752	2.5201726	1.00
Hybrid	8.76E-07	5.07E-07	**0.95**
Tetra-antennary	-1.32E-06	8.71E-07	**0.91**
High Mannose	-1.62E-07	2.14E-07	0.62
Triantennary	-2.19E-07	3.53E-07	0.50
Bisecting GlcNAcs	2.01E-07	1.82E-07	0.74
Biantennary	-4.71E-08	7.97E-08	0.49
Non-sialylated	-3.64E-08	6.05E-08	0.52
Non-fucosylated	4.13E-08	7.90E-08	0.42
Highly fucosylated	-3.52E-07	1.69E-06	0.58
Highly sialylated	2.51E-07	4.10E-07	0.42

With body weight

Term	Resampling Estimate	Resampling Std Dev	Percent Nonzero
Intercept	3.4397396	2.8325232	1.00
Hybrid	5.79E-07	4.64E-07	**0.87**
Tetra-antennary	-7.97E-07	9.14E-07	**0.71**
High Mannose	-9.14E-08	1.64E-07	0.50
Triantennary	-2.30E-07	3.04E-07	0.67
Bisecting GlcNAcs	1.31E-07	1.49E-07	0.67
Biantennary	-1.46E-08	3.90E-08	0.31
Non-sialylated	-1.44E-08	4.44E-08	0.47
Non-fucosylated	1.41E-08	4.48E-08	0.31
Highly fucosylated	-1.58E-06	2.25E-06	0.64
Highly sialylated	1.83E-07	3.26E-07	0.34
Weight	-0.033961	0.0117676	1.00

With body fat mass

Term	Resampling Estimate	Resampling Std Dev	Percent Nonzero
Intercept	-5.520276	3.0751797	1.00
Hybrid	8.92E-07	4.62E-07	**0.95**
Tetra-antennary	-8.27E-07	8.64E-07	**0.75**

High Mannose	1.84E-07	1.47E-07	0.81
Fat-mass	-0.028123	0.0123198	0.99
Triantennary	-4.54E-08	9.20E-08	0.36
Bisecting GlcNAcs	2.63E-08	6.50E-08	0.29
Biantennary	-2.36E-10	2.06E-08	0.19
Non-sialylated	2.10E-08	4.70E-08	0.39
Non-fucosylated	-1.46E-09	1.92E-08	0.18
Highly fucosylated	-6.20E-09	1.61E-06	0.47
Highly sialylated	3.88E-09	7.57E-08	0.17
